# Whole-Plant Water Use in Field Grown Grapevine: Seasonal and Environmental Effects on Water and Carbon Balance

**DOI:** 10.3389/fpls.2018.01540

**Published:** 2018-11-12

**Authors:** Cyril Douthe, Hipólito Medrano, Ignacio Tortosa, Jose Mariano Escalona, Esther Hernández-Montes, Alicia Pou

**Affiliations:** ^1^Research Group on Plant Biology Under Mediterranean Conditions, INAGEA, Department of Biology, University of the Balearic Islands, Palma, Spain; ^2^Instituto de Ciencias de la Vid y del Vino, Logroño, Spain

**Keywords:** grapevine, water use efficiency, whole plant chambers, water stress, carbon balance

## Abstract

Water scarcity is a main challenge in vineyards sustainability in most of the grapevine areas now and even more in near future due to climatic change perspectives. In consequence, water use efficiency (WUE) measurements are of the highest interest to improve the sustainability of this crop. The vast majority of WUE measurements relays on measurements of leaf carbon and water fluxes at leaf-level. However, less data are available at the whole-plant level, and for the moment those data are not totally coincident with conclusions reached at leaf scale. In this study, we used whole-plant chambers able to enclose an entire plant of 12 years old to measure at the same time water and carbon fluxes under realistic field grown conditions. The main objectives were to identify the technical issues interfering the whole-plant measurements and track the environmental and other abiotic factors that can affect water and carbon balance, i.e., WUE at the whole-plant scale. To achieve those objectives, we measured whole-plant water and carbon fluxes in grapevine exposed to two different water regimes at three phenological stages [pea size (July), ripening (August), and harvest (September)]. In September, measurements were repeated under high CO_2_ to also check its effect at the whole-plant scale. The results indicate that water and carbon fluxes are well coordinated under both water availability treatments. Under drought conditions, both fluxes were drastically reduced, but surprisingly the estimated WUE resulted not improved but decreased, contrarily to what is shown at the leaf scale. The phenology (September) also strongly decreased both water and carbon fluxes when compared to measurements in July. We hypostatized that harvest load respiration rates could have an important weight on the whole-plant net carbon exchange (NCE). Finally, high CO_2_ measurements, after correction for leaks, indicated an increase of whole-plant NCE as well as increased whole-plant WUE, as expected. Several technical issues were identified, like 1/instability of [CO_2_] during the night period that prevent robust estimation of whole-plant respiration and 2/condensation during last night and sun-rise hours which may affect the estimation of daily plant transpiration.

## Introduction

Water use efficiency (WUE) refers to the ratio of water used in plant carbon assimilation (photosynthesis; A_N_) or in biomass production to water lost by the plant through transpiration (E) and it has become an important parameter to take into account in agricultural systems to increase yield production in semi-arid areas to get more crop per drop. Either A_N_ or E are commonly recorded on single leaves by using portable infrared gas analyzers (IRGAs). However, in canopies such as grapevine and other crops, it has been shown that leaf-level photosynthesis measurements are largely dependent on leaf position (Escalona et al., 2003) so that typical single-leaf measurements can provide incomplete information if extrapolated to quantify photosynthesis at the whole-plant level. On the other hand, whole-vine photosynthesis expressed on a leaf area basis usually results below than expected extrapolating values from single-leaf measurements (Edson et al., 1993; Intrieri et al., 1997; Poni et al., 2009). Factors such as leaf light exposure and position on the shoot, leaf aging and the presence of organs like fruits, shoots, and trunks in a given canopy makes difficult to scaling up from single-leaf to whole-canopy photosynthesis (Alleweldt et al., 1982; Schultz, 1993; Intrieri et al., 1997; Poni et al., 1997; Petrie et al., 2000; Escalona et al., 2003). Moreover, other processes such as nocturnal water loss and respiration (Escalona et al., 2012, 2013) or possible changes in dry matter partitioning among different sinks (Tomàs et al., 2014) may explain a frequently reported lack of correlation between WUEi and WUE expressed as biomass accumulation per unit of water lost.

Scaling up to the whole canopy by using meteorological methods such as eddy correlation or covariance (Field et al., 1989; Long and Hällgren, 1989; Garcia et al., 1990), or by enclosing methods in open system flow-through chambers in which water vapor and CO_2_ fluxes are measured using an IRGA (Garcia et al., 1990; Poni et al., 1997) has been explored as a way to achieve a reliable measurement of the whole-plant gas-exchange measurement. Moreover, it has become an interesting tool to assess whole-plant responses to climatic change conditions, e.g., high CO_2_, water stress, high temperatures, etc. But on the other hand, one of scientist’s current concerns is to design a good enclosure system to minimize disturbance of the plant natural environment (Intrieri et al., 1997; Poni et al., 1997; Perez Peña and Tarara, 2004), i.e., to diminish errors associated with the “chamber effect.” Hence, even with a highly transparent cover, an enclosure increases air temperature and reduces incident radiation intensity in the canopy as well as gas exchange between the plant and the atmosphere (Perez Peña and Tarara, 2004) and, contrarily of what it happened when measuring WUE at the leaf-level, these studies reported that WUE at the whole-plant level was not higher on a daily basis in deficit-irrigated vines in comparison with well-watered plants, likely due to their higher respiration rates (Pagay, 2016).

On the other hand, studies about the quantification of the CO_2_ flows from different organs such as stems, roots and fruits along the vine vegetative cycle and the grapevine’s leaves maturity effects on the whole-plant carbon budgets are important but rather scarce. Several previous studies showed the contribution of grapevine clusters to the total carbon balance (Ollat and Gaudillère, 2000; Palliotti and Cartechini, 2001; Escalona et al., 2012; Hernández-Montes et al. unpublished data) and they reported a contribution of 10 and 18% to the carbon required for fruit development obtained by fruit photosynthesis and by the whole berry respiration, respectively. In this sense, it would be expected that a high rate of respiration during fruit ripening would greatly reduce daily net CO_2_ exchange rate (NCER). Miller et al. (1997) stated a rapid decrease of the NCER rate from, through harvest, in sharp contrast to the broadleaf chamber which showed no change in A_N_ rate per unit leaf area over the same period. Thus, to quantify the CO_2_ flows from each organ may contribute to calculate more accurately total plant carbon balances.

In this work, we have used an open framed, open-top, flow through chamber, according to Perez Peña and Tarara (2004) as has been previously reported in Escalona et al. (2016). A detailed evaluation of the daily whole-canopy NCERs and whole-canopy transpiration rates (E) have been done. Parameters such as nocturnal transpiration (E_night_) and canopy dark respiration (*R*_d_), were also considered to evaluate the whole-canopy WUE and to point out potential problems of integrating this data.

The goals of this study were: (i) to asses about leaks and difficulties encountered when measuring daily NCER and E to identify improvements needed for a more accurate estimation rates, (ii) measure daily whole-plant WUE for grapevines as affected by different irrigation treatments and high CO_2_, and (iii) to estimate the contribution of the grapevine’s leaves age and berry development to the total plant carbon balance along the vine phenology.

## Materials and Methods

### Plant Material and Site of Study

Measurements were conducted in summer 2017 in an experimental vineyard planted in 2009 with Grenache vines grafted in 110-Richter rootstocks, at the University of Balearic Islands. Vines were planted with 2.5 m between rows and 1 m between vines in a N–S orientation, and were submitted to two water regimes: (i) a moderate water irrigation (I) applying a crop coefficient of 0.5 of potential evapotranspiration calculated using Penman–Monteith equation, and (ii) non-irrigation (NI) (see Escalona et al., 2016 for details). Plants were irrigated twice a week from June to September with drips delivering 2 L h^-1^ placed at 0.6 m from each other.

**FIGURE 1:**
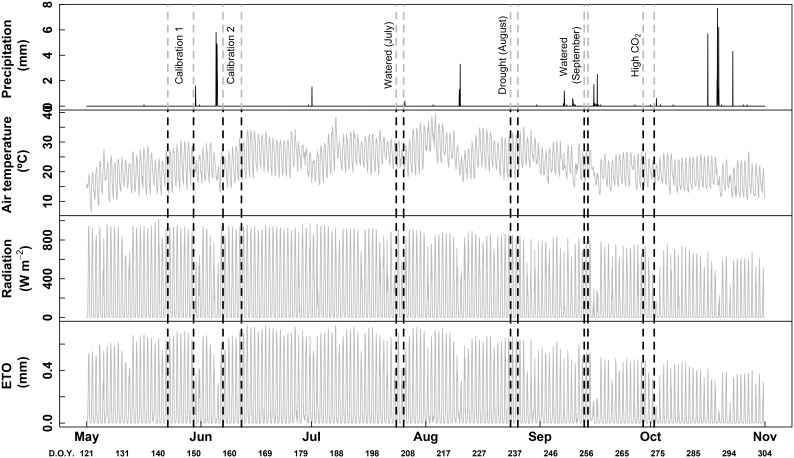
Hourly raw data of precipitation, air temperature, radiation and ET_0_ (potential evapotranspiration). Vertical dotted lines indicate dates of each session of measurements performed in this study, with the corresponding treatments. D.O.Y. for day of the year.

Measurements were carried-out in two representative plants for each phenological stage [pea size, irrigation (July); ripening, non-irrigation (August) and harvest, irrigation and high CO_2_ (September)]. The corresponding meteorological data is shown in Figure 1. The same irrigated plants were measured in July and September, while other two non-irrigated plants were measured in August. Thus, for pure phenological effect, sessions of July and September can be directly compared (same plants and both in watered conditions). For drought effects, sessions of August and September can be compared, since only 20 days separated the two measurements. For each session, plants remained inside the chambers 2 or 3 days before the measurements were taken to avoid the disturbing effect of the chamber installation. Afterward the chambers were removed and plants were grown outside the chambers until the next period of measurements (1 month between each period). Each measuring session (for each phenological stage) last two or three consecutive days. Concerning the data treatment, all data were averaged from 1 h.

### Meteorological Condition During the Experiment

Weather information was recorded using an automatic meteorological station located in the experimental vineyard (Meteodata, 3000, Geonica) (see Table 1 and Figure 1). Effective rainfall was <5 mm in July and August with similar mean diurnal temperatures (29.5°C) and relative humidity (46–48%), while measurements in September were performed with more rainfall (11.5 mm), lower mean diurnal temperatures (23.8°C) and higher relative humidity (67.1%).

**Table 1:** Average values of air temperature (T_air_, °C), relative humidity (RH, %), wind speed (m s^-1^), cumulative precipitations (Precip.), average radiation (Rad, W m^-2^) and cumulative evapotranspiration (ET_0_, mm) for each month of measurement and separated for day/night time.

Month	Daytime	T_air_	RH	Wind speed	Precip.	Rad	ETO
May	Day	23.23	46.83	1.43	2.6	541.21	136.62
June	Day	27.84	45.37	1.42	13.4	567.07	152.53
July	Day	29.49	46.65	1.37	0.4	531.70	152.53
August	Day	29.73	48.17	1.22	3.5	451.41	132.26
September	Day	23.78	67.10	1.14	8.1	353.73	88.28
October	Day	21.01	79.50	0.64	20.9	266.04	61.93
May	Night	15.62	75.25	0.23	0	0	0
June	Night	20.58	70.74	0.20	15.5	0	0
July	Night	22.50	71.00	0.18	1.5	0	0
August	Night	23.27	72.38	0.26	3.6	0	0
September	Night	18.42	88.41	0.19	3.4	0	0
October	Night	15.23	98.20	0.04	15.5	0	0

### Whole-Plant Gas Exchange Chambers

Whole-plant net carbon exchange (NCE) and transpiration were measured using two open-top chambers (3.36 m^3^ each) covered with plastic film (RX 140-Propafilm^TM^). An air flow (Series 641 Air Velocity Transmitter, Dwyer, IN, United States) through the chamber of 278 mol min^-1^ (F) was maintained for all measurements, delivered by a constant speed turbine (S & P 500) fed by a ∅ = 165 mm pipe taking the atmospheric air at 3 m above ground. Air entering and leaving the chamber was pumped (TD4x2 type NA; Braislford Pumps, United States) at a flow of 0.5–1 L min^-1^ to feed a calibrated gas analyzer (Li-840, Li-Cor, Inc., Lincoln, NE, United States). The air flow entering and outgoing the chamber was measured during five consecutive minutes each, alternatively. The four first minutes of data were eliminated to ensure a complete turn-over of the gas in the measuring circuit. [CO_2_] and [H_2_O] were used to estimate plant NCE and transpiration (E) with:

$$\begin{array}{l} \text{NCE}=\text{F}({\text{C}}_{\text{e}}-{\text{C}}_{\text{o}})/{\text{L}}_{\text{a}}\\ \text{ E}=\text{F}({\text{W}}_{o}-{\text{W}}_{e})/{\text{L}}_{\text{a}} \end{array}$$

where F is the air flow through the chamber, C_e_ and W_e_ the [CO_2_] and [H_2_O] entering the chamber, C_o_ and W_o_ the [CO_2_] and [H_2_O] outgoing the chamber and L_a_ the total leaf area of the plant. Plant leaf area was estimated in each one of the measured plants at harvest using the methodology proposed by Sánchez-de-Miguel et al. (2010).

Air temperature was measured with type K thermocouple (RoHS, Model TP-01), and placed in the top-center of the chamber. The atmospheric air temperature was recorded with a meteorological station (see above) situated at 50 m from the chambers. The difference in temperature inside/outside the chamber was comprised between 4 and 5°C, with some punctual peaks at 10°C during august. Some of these variations come from the fact that the thermocouple was directly exposed to sun (not shaded), which could increase the estimated temperature from the real one.

### Validation of the Gas Exchange Measurements

We intended to confirm the reliability of the gas-exchange measurements with an external estimation of the plant transpiration. For these measurements, we placed one potted vine in each chamber (total plant leaf area around 0.5 m^2^), placed on a balance (Baxtran, equipped with Giropes module L6E) to measure Transpiration by the loss of weight through 4 days. Chambers were equipped with the gas-exchange system described just above (except the 3 m chimney that was not placed for the calibration), and transpiration via gravimetry and gas-exchange were compared.

### Statistical Analysis

All the data analysis was performed using R (R Core Team, 2016), Foundation for Statistical Computing, Vienna, Austria).

## Results

### Conditions During the Study

The Figure 1 shows the meteorological conditions all along the experiment as well as the location of each session of calibration and measurement.

Climate conditions during the experiment (May–September 2017) were typical for Mediterranean regions, with daytime temperatures above 25°C, night temperatures above 20°C and diurnal radiation frequently reaching 800 W m^-2^ (Figure 1). Peak photosynthetic photon flux density (PPFD) at midday was usually 1500–1700 μmol m^-2^ s^-1^ (not shown).

### Comparison Between Gas-Exchange and Gravimetry

**FIGURE 2:**
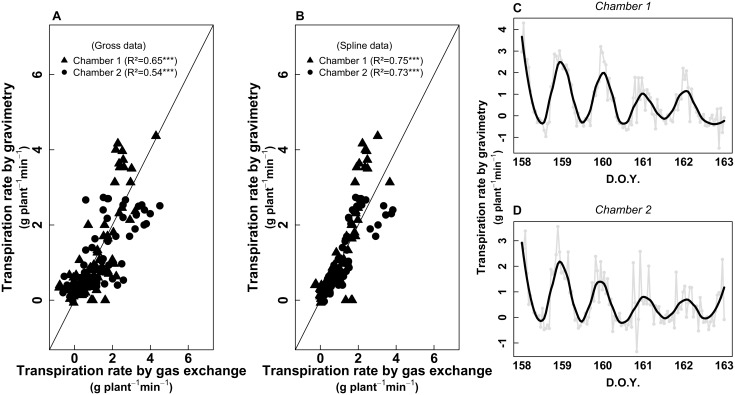
**(A)** Relationship between Transpiration rate (g H_2_O plant^-1^ min^-1^) estimated from gravimetry and gas exchange (whole-plant chamber). The continuous line represents the 1:1 relationship. **(B)** Same relationship but with data treated with a spline. The continuous line represents the 1:1 relationship. **(C,D)** gross data of gravimetry [gray circles and lines, same as presented in **(A)**] for chambers 1 and 2, respectively. The black lines represent the data treated with a spline in order to remove non-biological noise, as presented in **(B)**. The data in **(A,B)** come from two sampling sessions (first session: from D.O.Y. 143–150; second session: from D.O.Y. 158–163; see Figure 1).

To validate the gas-exchange measurements, a potted plant on a balance was placed in each chamber. The weight of each plant was monitored continuously during 24 h for five consecutive days. The calibration procedure was carried-out two times in May and June 2017 (see Figure 1). The resulted correlation between both estimations of the plant transpiration for chamber 1 and chamber 2 were *R*^2^ = 0.54 and 0.65 (both *p* < 0.001), respectively (Figure 2). We observed that gravimetry and gas-exchange data were sometimes noisy (irregular peaks). Such peaks were not related to plant transpiration dynamics but to 1/noise measurement form the device and 2/some possible condensation inside the plant chamber. Diurnal cycles were treated with a spline to eliminate such non-biological noise (Figures 2C,D). The resultant correlations were improved for both chambers (Figure 2B), with *R*^2^ = 0.75 and 0.73 (both *p* < 0.001) for chamber 1 and 2, respectively. We also observed a progressive decrease of the maximum transpiration rate along the calibration session (5 days), since plants were not watered while being inside the chamber.

### Technical Limitations for the Measurements

**FIGURE 3:**
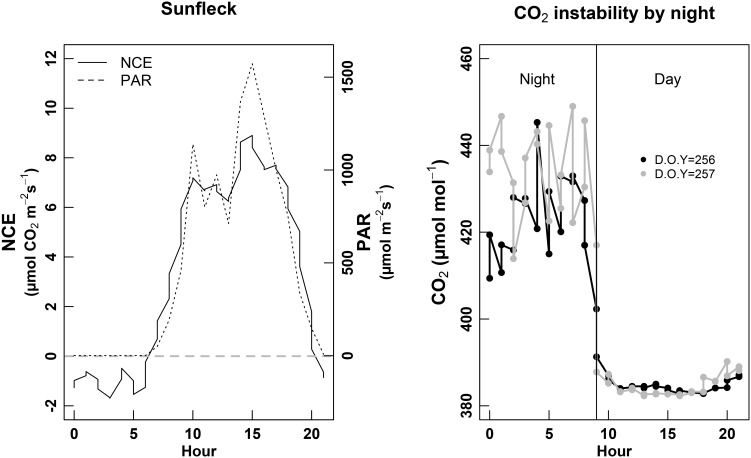
Left panel: example of sun fleck (PAR, discontinuous line) effect on the NCE (net carbon exchange of the whole-plant, continuous line). Right panel: monitoring of the [CO_2_] entering the chamber (atmospheric [CO_2_]) over a complete 24 h cycle, during two consecutive days (D.O.Y. 256 black line and D.O.Y. 257 gray line). The vertical continuous black line denotes the day/night limit.

We identified some of the limits encountered when using whole-plant chambers. First, the atmospheric (entering air flow) [CO_2_] was instable during the night (Figure 3, right). While the day [CO_2_] was around 380 μmol mol^-1^ and presented very low hourly variations along the day (∼5 μmol mol^-1^), [CO_2_] used to increase up to 430 μmol mol^-1^ during the night, with 20–30 μmol mol^-1^ variation between two consecutive hours. The same variation pattern was observed for both chambers, and for each daily cycle measured during the session of September (harvest), but these variations were less pronounced than during the other two sessions. In parallel, we observed that NCE estimates during the night were very noisy; meanwhile during the day they were much more robust. We also observed that this gas-exchange system was able to pick the effect of sun-flecks on NCE. Indeed, PAR variations between 700 and 1,500 μmol photons m^-2^ s^-1^ within 30 min—1 h (punctual clouds) could induce variations of NCE between 6 and 8 μmol CO_2_ m^-2^ s^-1^ (Figure 3, left).

**FIGURE 4:**
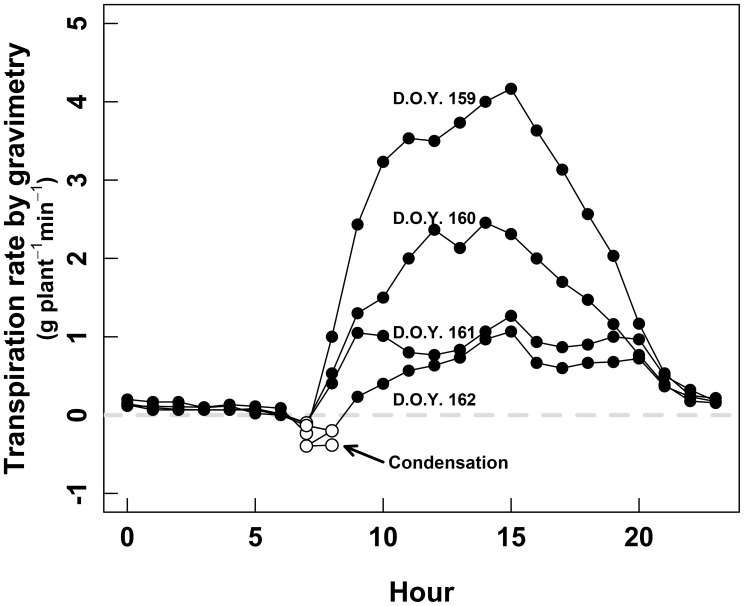
Daily course of the whole-plant transpiration measured by gravimetry during the second calibration session (D.O.Y. 158 to D.O.Y. 162). The black dots denote positive transpiration, while the white dots denote negative transpiration, interpreted as condensation during the earlier hours of the day.

The whole-plant gas-exchange system also allowed us to detect condensation at the sun-rise. This was demonstrated by negative transpiration, systematically occurring between 6 and 9 h in the morning. This phenomenon was observed either by gravimetry (Figure 4), and gas exchange (not shown).

### Carbon and Water Balance Through Different Phenological and Environmental Conditions

**FIGURE 5:**
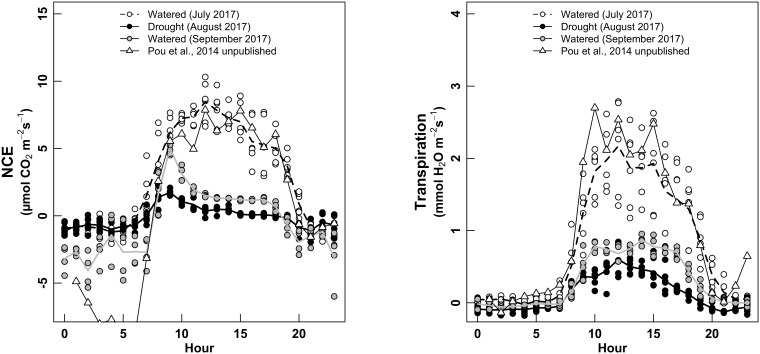
Daily cycle of NCE—whole-plant net carbon exchange (left panel, μmol m^-2^ s^-1^) and whole-plant Transpiration (right, mmol m^-2^ s^-1^), for three water treatments: white dots for “Watered” (measured in July 2017); black dots for “Drought” (measured in August 2017); gray dots for “Watered” (measured in September 2017). The white triangles are measurements performed in 2015 (Pou et al., 2014, unpublished), using the same chamber and experimental set-up in watered conditions.

Gas-exchange rate was clearly affected by phenological period of the plant, as expected. When measured in July (pea size, irrigated plants), plant NCE described a classical Gauss curve along the day (Figure 5, left), with the maximum peak reached at 12 h and NCE values of 10 μmol m^-2^ s^-1^. The whole-plant transpiration (E) described the same type of variations, with maximum values of 2.5–3 mmol H_2_O m^-2^ s^-1^, coordinately with the peak of NCE (Figure 5, right).

When measured during the ripening (August, non-irrigated conditions), vines described a different daily cycle, with the maximum NCE of 1–2 μmol m^-2^ s^-1^ around 8–9 h in the morning, then decreasing slowly during the day (Figure 5, left). E was also strongly decreased, with maximum values of 0.5 mmol m^-2^ s^-1^ around 12–13 h (Figure 5, right). We observed that the non-irrigation conditions in August provoked a dramatic decrease in absolute values of both NCE and E, but with a strong asymmetry along the day for NCE (taking advantage of the early morning) but not for E.

During the harvest period (irrigated conditions, before harvesting the fruits), both NCE and E described the same shape of daily cycle than under non-irrigated condition (August, i.e., with an asymmetric for NCE, not for E), with clearly lower values than for the measurements during July (irrigated conditions), but higher than in August. The night respiration was much more erratic and higher (more negative values) during the harvest (September, irrigated conditions, with higher fruits load) than during previous periods. Finally, we checked whether NCE values of our study were concordant with values previously measured by Pou et al. (2014, unpublished), using the same gas-exchange system, in watered conditions and for same grape cultivar (Grenache). Absolute values and shape of both NCE and E along the day were identical with the measurements of this previous study. The only differences were shown for night respiration, higher (more negative) for Pou et al., 2014, unpublished) than this study. No relationship was found between temperature and night NCE (not shown).

### Light Intensity and Carbon and Water Balance Under Different Phenological and Environmental Conditions

The measurements performed during full daily cycles, enabled to perform a comparison of photosynthesis and transpiration dependency from incoming light. This allowed to establish light response curves of NCE and E under different phenological and environmental conditions.

**FIGURE 6:**
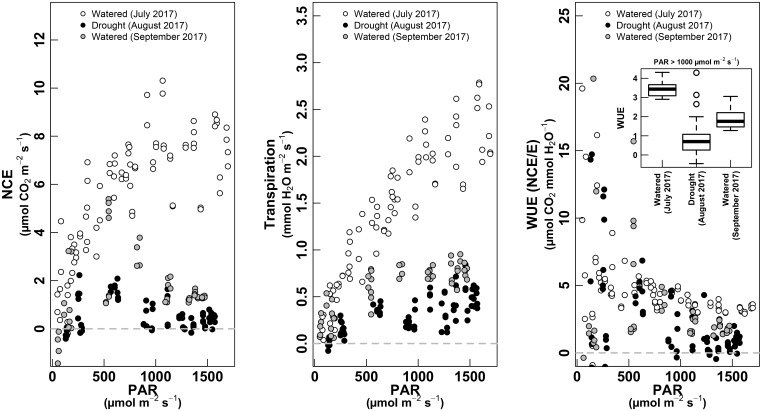
PAR response curves for NCE—whole-plant net carbon exchange (left panel, μmol m^-2^ s^-1^), whole-plant Transpiration (middle panel, mmol m^-2^ s^-1^) and WUE as NCE/E (right panel, μmol CO_2_ mmol^-1^ H_2_O). The relationship is shown for the three water treatments: white dots for “Watered” (measured in July 2017); black dots for “Drought” (measured in August 2017); gray dots for “Watered” (measured in September 2017). The boxplot inset in the right panel shows values of WUE for each treatment when PAR is >1,000 μmol m^-2^ s^-1^. Only data with PAR >50 μmol m^-2^ s^-1^ are shown.

During the pea-size period (July, irrigated conditions), both NCE and E described a positive relationship with the increase in light through the day. NCE followed a clearly saturating shape at high light (after ∼1,000 μmol m^-2^ s^-1^), while E described a less saturated response (Figure 6, left and middle).

The NCE and E response to light showed lower values under non-irrigated conditions (August, ripening). NCE showed an asymmetric response, with maximum values reached at ∼500 μmol m^-2^ s^-1^ and with a clear decrease at highest light intensity. The E response in August was not asymmetric, with a classical saturated response to light (Figure 6, middle). Again, we observed that during the harvest (September, irrigated conditions), NCE and E showed the same shape of daily cycle as under non-irrigated conditions (August), but with slightly higher absolute values.

The WUE described a decreasing curvilinear tendency with increasing light, with high variations at low light, corresponding to sunrise and sunset (Figure 6, right). In both irrigated conditions (especially in July, with well performing plants), the WUE was higher than under non-irrigated plants (see inset in Figure 6, right).

### Response to High CO_2_ Conditions

**FIGURE 7:**
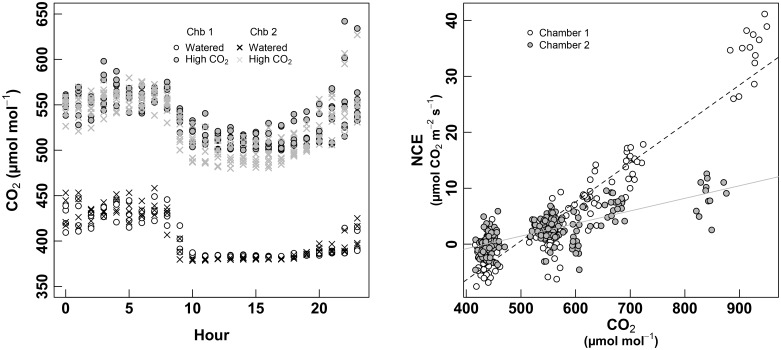
Left panel: monitoring of the [CO_2_] entering the chamber over cycles of 24 h. White dots denote the second “Watered” session (September 2017) for the chamber 1, and the black crosses for the chamber 2, both of them at ambient (atmospheric) CO_2_. Gray circles and gray crosses denote the “High CO_2_” treatment (September 2017) for chambers 1 and 2, respectively. Right panel: calibration curves used to correct the estimated NCE during the “High CO_2_” experiment. White dots are for the chamber 1, and gray dots for the chamber 2. Measurements where carried-out during the night to avoid photosynthetic processes to influence the measurements (and considering the night-respiration as negligible), then [CO_2_] was increased by steps from 400 μmol mol^-1^ to approximately 900 μmol mol^-1^ in order to obtain a calibration curve. This curve was then subtracted to the row-data obtained under high CO_2_ conditions.

At the end of the measurement session of September 2017 (in watered condition and pre-harvest period, see Figure 1), instead of removing the plants from the chambers, they were kept inside and [CO_2_] entering the chamber was increased until a level of 500 μmol m^-2^ s^-1^ (Figure 7, left). Plants were kept 5 days in high CO_2_ conditions before taking measurements. Calibration curves were also performed before analyzing the raw data (Figure 7, right). After this pretreatment, the whole-plant CO_2_ response was studied increasing steep by steep the CO_2_ concentrations inside the chamber (from 400 to 900 ppm).

**FIGURE 8:**
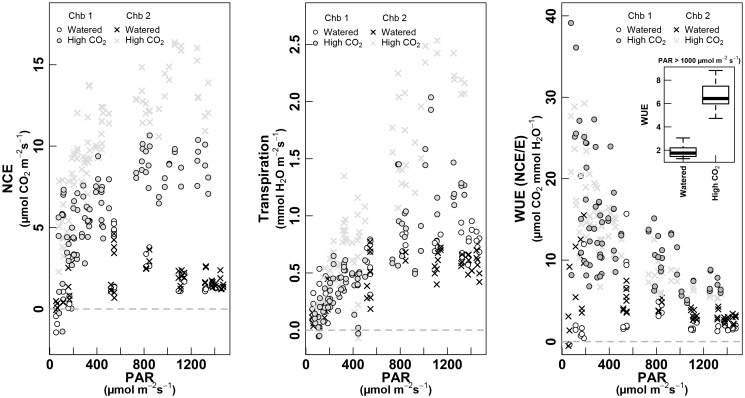
PAR response curves for NCE—whole-plant net carbon exchange (left, μmol m^-2^ s^-1^), whole-plant Transpiration (mmol m^-2^ s^-1^, middle) and WUE as NCE/E (μmol CO_2_ mmol^-1^ H_2_O, right). The relationship is shown for the two treatments: white dots for “Watered” of chamber 1 and crosses for chamber 2; gray dots for “High CO_2_” for chamber 1 and gray crosses for the chamber 2 (both treatments were measured in September 2017, on the same plant). The boxplot inset in the right panel shows values of WUE for each treatment when PAR is >1,000 μmol m^-2^ s^-1^. Only data with PAR >50 μmol m^-2^ s^-1^ are shown.

Each chamber showed a different response of apparent NCE to [CO_2_], likely due to different intensity of leaks (see Figure 7, right). Nevertheless, the total pool of NCE data showed a continuous relationship to [CO_2_] when plotted against corrected-NCE (Figure 8, left).

Under high CO_2_, the NCE along the day was clearly higher than under ambient [CO_2_], measured 5–6 days before (Figure 8, left). While plants in ambient [CO_2_] showed a strong decrease at highest PAR intensities, under high [CO_2_] they maintained a high NCE (∼10 μmol m^-2^ s^-1^) along the whole day. The chamber 2 showed slightly higher values than chamber 1, but keeping in the same range, and recovering both the values of watered condition in July. Surprisingly, the values of transpiration increased under high CO_2_ when compared to previous values of watered conditions in September, but fitted very well with *E*-values of July, in watered conditions (Figure 6, middle). The WUE was clearly increased under high CO_2_ (Figure 6, middle), four by eightfold when compared to September in watered conditions, and by twofold when compared to July, watered conditions (see inset Figure 8, right).

## Discussion

A better understanding of Carbon and water fluxes at the whole-plant scale is needed to improve the natural ecosystems and crops water management. But this must be done firstly by improving the knowledge of the relationship between whole-plant and single-leaf gas exchange, and their occasional discrepancy when they are directly compared (Medrano et al., 2012; Tomàs et al., 2012). In particular, when talking about WUE, the variability of leaf to leaf environmental conditions and plant gas-exchange regulation could lead to some contradictory results (Poni et al., 2009; Tarara et al., 2011; Escalona et al., 2016). To understand why intensive leaf-level measurements do not always completely reflect the whole-plant physiology, an exhaustive array of data was gathered using entire enclosed plants. To ensure reliability of the data from the whole-plant gas-exchange system, a preliminary calibration was set in order to calibrate chamber-derived transpiration rates by comparing gas exchange versus gravimetric vine water loss. The resulted correlations between both estimations of the plant transpiration were sometimes noisy and apparently not related to plant transpiration dynamics. Thus, diurnal cycles were treated with a spline to eliminate such non-biological noise leading to close linear relationships (*R*^2^ = 0.75 and 0.73; both *p* < 0.001 in chamber 1 and 2, respectively). This resulted in a successful validation of the chamber-derived vine transpiration, in agreement with findings previously reported by Poni et al. (1999, 2014). These noisy measurements faced two large problems. The first one was probably due to the absence of the 3.5 m metal pipe during the calibration process, usually added to minimize fluctuations in ambient CO_2_ (Perez Peña and Tarara, 2004), emphasizing the importance of having a proper buffer volume during measurements. The second was likely derived from a detected condensation, both by gravimetry and by gas exchange, which systematically occurred between 6 and 9 h a.m., when the temperature of the inner surface of the chamber was surely below the dew point. Maintaining a minimum temperature increase in the chamber and improving air circulation inside whole-plant chambers is therefore necessary to avoid condensation when calculating daily transpiration rates.

Moreover, tracking the whole-plant carbon balance during the vine growing cycle was attempted to be verified by firstly checking the flux of [CO_2_] during night periods (i.e., nocturnal NCE or night respiration, *R*_d_). Surprisingly, [CO_2_] measurements at night stand out for their instability, which were especially high during harvest period and not dependent of wind speed or other environmental factors. Such [CO_2_] instability has been previously described by Patil et al. (2014), who attributed the pronounced CO_2_ peaks to the absence of solar radiation and accumulation of CO_2_ in unstable boundary layer. Thus, these noisy measurements may lead to unreliable night respiration estimations, again reinforcing the idea that proper buffer volumes are necessary when large plant chambers are used.

### Carbon and Water Balance Through Different Phenology and Environmental Conditions

Instantaneous rates of NCE showed reliable and stable values, and varied with irradiance, temperature and elapsed time, as expected. Over the course of the day, NCE highly correlates with canopy light interception, as also have been reported by Petrie et al. (2009) and Poni et al. (1997). Daily cycles of NCE were very similar for the two water regimes and the two different phenology stages in early morning and late afternoon when sunlight was largely diffuse. Conversely, canopy NCE trends started to differentiate and showed maximum difference from about 9 till 16 h when canopies were subjected to a higher proportion of direct light. Thus, we stressed that the daily NCE measurements were affected by the applied water regime, both pre- and post-véraison, obtaining different shapes when comparing between irrigation treatments (July vs. August) and between different phenological times (different aging) (July vs. September), even being under the same water regime. NCE for watered plants (in July) followed the sinusoidal pattern of irradiance. By contrast, in August (for stressed plants) and September (for watered plants), instantaneous NCE reflected a morning maximum (8 h) peak of about 5 and 2 μmol m^-2^ s^-1^, respectively, and then declined steadily after 12 h. Most of the higher CO_2_ fixation rates during the entire day in watered plants in July could be due to the faster canopy development (high proportion of fully expanded leaves) rather than a higher average photosynthetic rate per leaf, as it has been previously described by Poni et al. (2000). On the other hand, instantaneous rates of NCE in August reflected a combined effect of water stress (no irrigation delivery), similarly of what has been previously observed in Tarara et al. (2011). However, a different trend was observed by Pagay (2016), who did not show differences in diurnal NCE patterns pre-veraison under different water regimes. This was likely because vines did not have a sufficiently long period without irrigation to experience water deficit.

Interestingly, *R*_d_-values in the present study showed slight decreases between pea size (in July) vs. pre-harvest (in September), within the same irrigation level. The dearth of information on *R*_d_ as affected by water stress and leaf aging makes comparisons with the present study difficult. On one side, Pagay (2016) described higher *R*_d_-values for deficit-irrigated vines, however, in our experiment, the system sensitivity might not be sufficient to highlight early-season differences in respiration with respect to different irrigation regime mainly due to the obtained high nocturnal CO_2_ fluctuations and the impossibility of adjusting flow rates at night. On the other side, Zufferey (2016) and Hernández-Montes et al. (2018) showed that during the rapid plant growth phase, young leaves presented the highest *R*_d_ rates compared to the mature ones. However, once the vegetative growth had stopped, the effect of the leaf age on *R*_d_ was less noticeable. At this stage (i.e., when the vegetative growing phase stops), we can hypothesized that the contribution of grapevine clusters to the total carbon balance by the whole berry respiration, might be highly enough to greatly reduce the daily NCE. Accordingly, Miller et al. (1997) stated a rapid decrease of the NCE rate from veraison through harvest.

Moreover, recent results showed that bunches respiration at veraison and ripening are the main component of plant respiration (Hernández-Montes, 2017), so that this component can be an important part of the NCE rates of the whole canopy during those months reducing significantly the net carbon uptake. On the basis of this important CO_2_ flux, a more detailed study of the influence of the grapevine clusters to the total NCE is necessary to better understand the whole-plant carbon fluxes.

Patterns of canopy transpiration (E) followed a similar outline than VPD and PAR (data not shown). In accordance to Tarara and Pena (2015), similar patterns of E were obtained between irrigation regimes, and between time points. However, it is noticeable here, that all along the entire experiment, different absolute values of *E* and *E*_night_ (nocturnal water loss) varied following both, different water regimes and the leaf aging of the plant. On the one hand, either at leaf and whole-canopy level, it is well documented that the most important environmental factors affecting transpiration are humidity, temperature, light intensity, wind, and the soil water content (Escalona et al., 2003; Medrano et al., 2003; Merli et al., 2015; Pagay et al., 2016). However, on the other hand, less attention has been given to the evolution of whole-plant transpiration throughout the vine vegetative cycle. Boyer et al. (1997) described the importance of leaf aging in the *E* levels, mainly because of a great reduction of the cuticular transpiration. So, at the whole-plant level and during the harvest stage, it was expected to have decreased *E*-values, as it happens here (Figure 5B). It can be also hypothesized that the decrease of photosynthetic capacity due to leaf ontology through the season goes along a decrease in *E*.

### Responses to Light

Over the course of the day, studies have found that patterns of NCE correlates highly with canopy light interception (Poni et al., 1997; Petrie et al., 2009). Pooling data gathered throughout the different treatments permitted to obtain different light response curves for NCE and *E*. The obtained curves were similar to that known for the light dependence of CO_2_ assimilation (Harbinson and Foyer, 1991). However, compared with single-leaf light response curves, whole-canopy photosynthesis in well-watered plants showed a much more gradual gain in NCE with increasing PAR and lack of a clear saturation plateau (Corelli-Grappadelli and Magnanini, 1997; Intrieri et al., 1997; Poni et al., 2003).

At low light intensities, below 500 μmol photons m^-2^ s^-1^, similar values of NCE and *E* were observed for the two watered treatments (July and September data) but at high light intensities the maximum values decreased with leaf aging. Indeed, since light intensity is coupled to the energy balance of leaves, the light response curves of photosynthesis are modulated by leaf age through differences in the stomatal sensitivity, and also boundary layer conditions (Field and Mooney, 1983; Zufferey et al., 2000; Poni et al., 2003), likely leading to obtain such differences on a seasonal basis. Additionally, because high light intensity hours are in coincidence with high temperatures, their effect on whole-plant respiration and mainly clusters respiration surely influence the observed light curve shape.

The light response curves of photosynthesis became distinctly flatter with increasing water scarcity. In this case, there was a significant treatment effect starting at low irradiance. Moreover there was a lack of a sharp saturation threshold in the trends of *E*, which continued to increase even after NCE had started to decrease.

Regardless of the amount of the water supplied and the leaf aging effect, canopy WUE exhibited decreasing trends and flattened out from approximately 500 μmol photons m^-2^ s^-1^. Contrarily to the expected, canopy WUE was distinctly higher in the well-watered treatment than under water stress. This scenario differed from that which might derive from traditional single-leaf assessment and some other estimates on the basis of single-leaf measurements made in the entire canopy (Escalona et al., 2003; Medrano et al., 2012). However, these results are in line with most of studies which have tackled the combination of both, leaf and whole-canopy gas exchange (Poni et al., 2009, 2014; Tarara et al., 2011; Merli et al., 2015). Only Palliotti et al. (2014) reported that drought vines exhibited increased WUE at both the single-leaf and whole-canopy levels on *Vitis vinifera* cv. Sangiovese. Supporting our results, Intrieri et al. (1998) has shown that shaded or partially shaded leaves usually show lower WUE than well-exposed ones since low light limits photosynthesis more than water loss. So, in this case, scaling up WUE readings from leaves to whole-plant, lead to a disappointingly low correlation, mainly because of the obvious differences that a whole canopy may present in terms of leaf exposure and the dynamics of light interception during the day. Moreover, the recent results on the respiration rate of bunches throughout this period (véraison-ripening), could contribute to better understand the discrepancy among single-leaf and whole-canopy results. So, the overall carbon gain is ultimately regulating total plant growth and thus, the study of respiration and photosynthesis of intact whole clusters under field conditions can led us to gain important new knowledge about the real contribution of fruit to the total vine carbon balance.

### Responses to High CO_2_

Increases of atmospheric CO_2_ concentrations are rising year by year at a rate that has been shown to affect photosynthetic rates in C3 plants (Gerhart and Ward, 2010). There is no doubt that growth at elevated [CO_2_] stimulates NCE in C3 plants (Drake et al., 1997; Norby et al., 1999; Nowak et al., 2004; Ainsworth and Long, 2005; Ainsworth and Rogers, 2007). We conducted different response curves of NCE to increasing [CO_2_], and as predicted, there were significant and marked increases in NCE with increasing [CO_2_]. However, the relative rises in NCE were steeper at low irradiance probably because ATP concentration responds more steeply to increasing CO_2_ supply when photosynthesis is limited by RuBP regeneration (Buckley et al., 2003).

When comparing between the two analyzed plants for the same treatment, we have obtained similar shapes of NCE to the increasing [CO_2_] values, however, at high CO_2_, curves were shifted to higher NCE values for chamber 2. This difference would not be explained by the “chamber effect,” as similar [CO_2_] was entering in both chambers (Figure 7), so, a much further effect to high CO_2_ may be suggested in this case for the enclosed plant 2.

Concerning the PAR response curves for transpiration to rises in [CO_2_], and contrary of what was expected (Long et al., 2004) increasing transpiration values in response to increasing [CO_2_] have been obtained for the two enclosed plants. We may argue that other direct effects on water loss by transpiration such as ambient temperature, VPD or other driving forces for exchange of the water vapor from the leaf surface to the surrounding atmosphere may even dominate over the stomatal conductance, but also a differential CO_2_ effect on g_s_ for the shaded canopy leaves. Also, plants were subjected to high [CO_2_] during 3–4 days before taking the measurements, while the single-leaf CO_2_ response are always performed at the minute scale. A medium-term stomatal adjustment could also explain this higher *E* under high [CO_2_] conditions. However, a complication for the correct estimation of the effect of elevated [CO_2_] on transpiration is that experiments have been performed in environmentally controlled and generally well mixed and ventilated experimental set-up (open-top chambers), where the indirect effects may not show up so prominently as in a real and outside future climate (Unsworth et al., 1984; Leuning and Foster, 1990).

## Conclusion

Whole-plant chambers were developed as a mean to measure gas-exchange rates in grapevines growing in the field, thus becoming a valuable way to handle the daily variations of carbon and water fluxes in the whole-plant under variable environmental conditions [i.e., water regimes, (CO_2_), etc.], and leaf aging.

These results clearly show marked differences in daily NCE and *E* all along the vine vegetative growing cycle as well as when comparing between irrigation treatments.

Water scarcity and leaf aging were shown to significantly decrease daily water use but at expenses of lower NCE, but reducing WUE and confirming the lack of correlation between WUEi and whole-canopy WUE. At late stages (i.e., when the vegetative growing phase stops), we suggest that the contribution of grapevine clusters to the total carbon balance by the whole berry respiration, might be high enough to greatly reduce the daily NCE, and thus to ultimately regulate total plant WUE.

Future studies in this area should address to measure the specific contribution of grapevine clusters to the whole-plant NCE as it may explain the reported discrepancy between WUEi and whole-canopy WUE.

## Author Contributions

All authors contributed to the experiments, data collection, and results evaluation. HM supervised and discussed the manuscript.

## Conflict of Interest Statement

The authors declare that the research was conducted in the absence of any commercial or financial relationships that could be construed as a potential conflict of interest.
